# Supplementary material for: PARP-1 Expression and BRCA1 Mutations in Breast Cancer Patients’ CTCs
Source: Cancers (Basel). 2022 Mar 29;14(7):1731. doi: 10.3390/cancers14071731 (PMC8996866; doi:10.3390/cancers14071731)
Supplement: Supplementary file 1 [file cancers-14-01731-s001.zip › cancers-1638455-supplementary.pdf]

cancers

Supplementary Materials

PARP-1 Expression and *BRCA1* Mutations in Breast Cancer Patients' CTCs

Thodoris Sklias, Vasileios Vardas, Evangelia Pantazaka, Athina Christopoulou, Vassilis Georgoulias, Athanasios Kotsakis, Yiannis Vasilopoulos and Galatea Kallergi

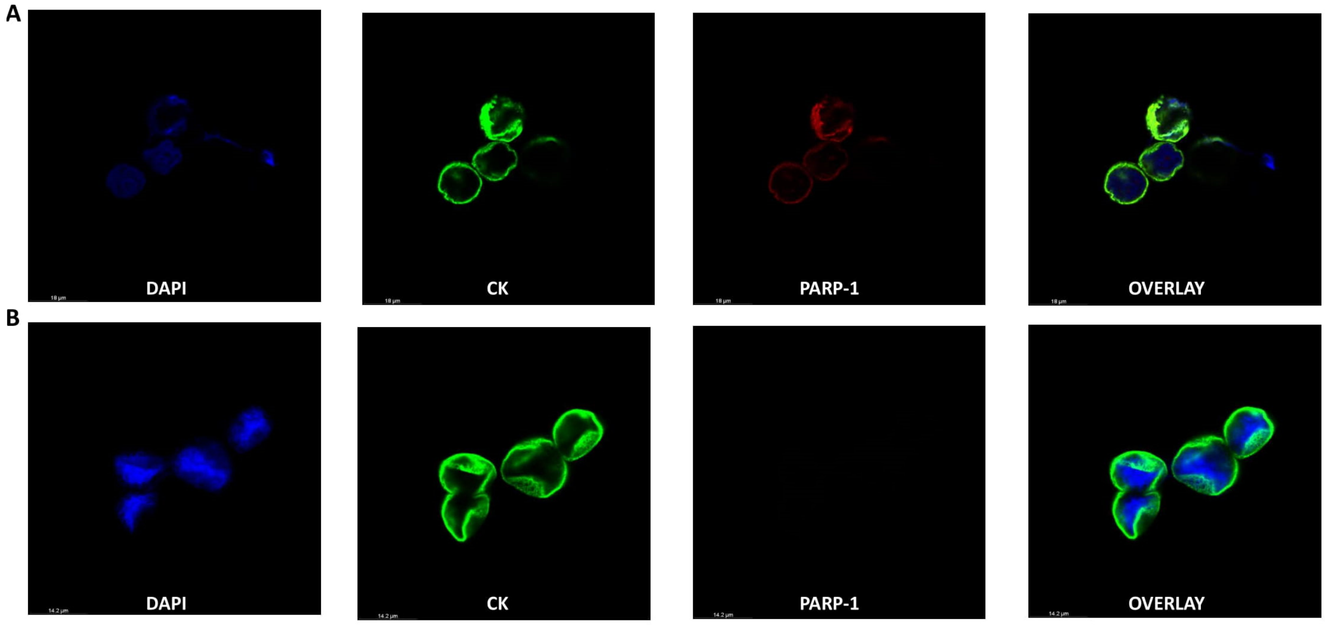

Figure S1. Representative confocal laser scanning micrographs of the positive and negative controls of CK/PARP staining. (**A**) MCF-7 cells, spiked in normal blood and isolated with Ficoll-Hypaque, stained for CK (green) and PARP (red; positive control); (**B**) Cells stained for CK (green; negative control for PARP).

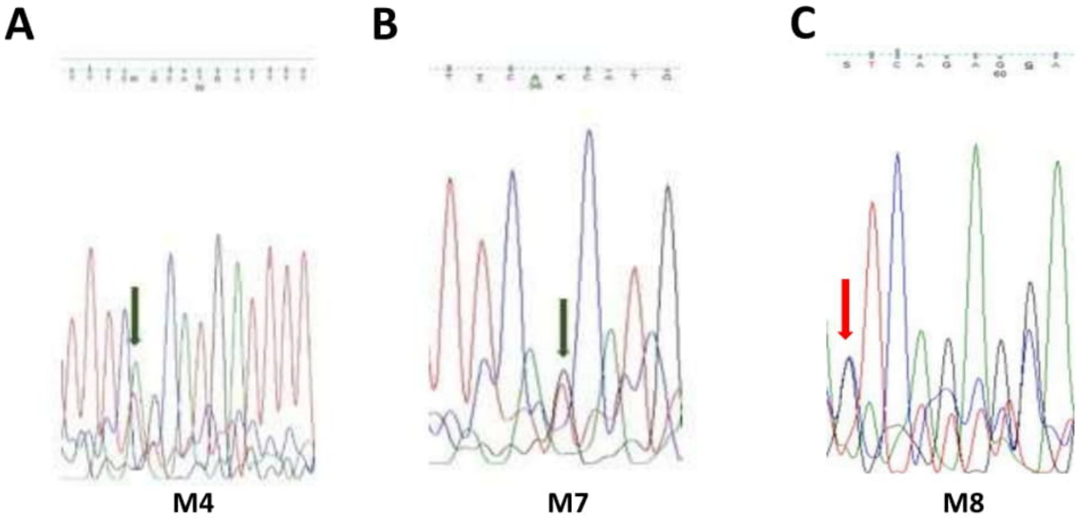

Figure S2. Representative chromatographs of the detected pathogenic mutations (**A**) M4; (**B**) M7 and (**C**) M8.

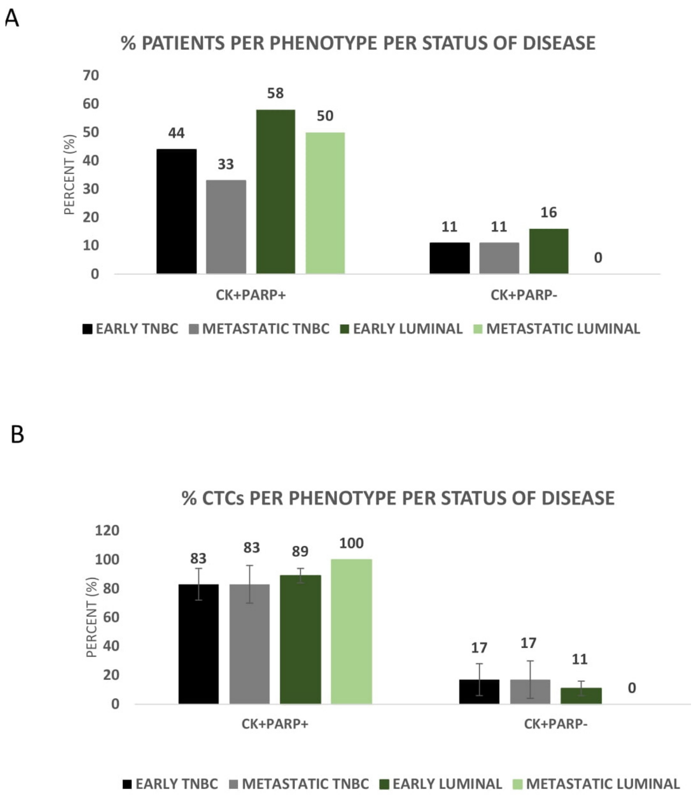

Figure S3. PARP-1 expression in TNBC and luminal BC patients' CTCs. (**A**) Percentage of early and metastatic TNBC and luminal patients with CK^+PARP^+ or CK^+PARP^- phenotypes; (**B**) Percentage of CTCs with CK^+PARP^+ or CK^+PARP^- phenotypes in early and metastatic TNBC and luminal patients.

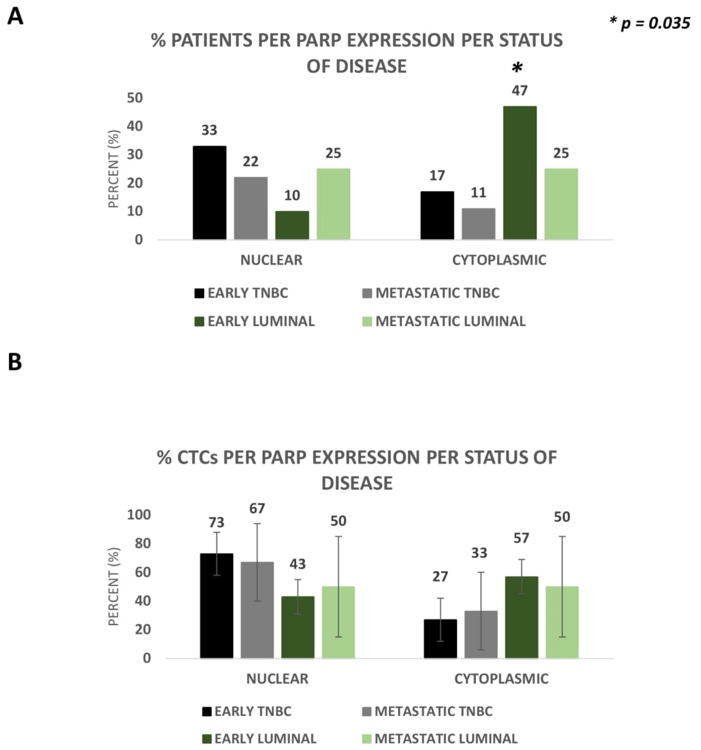

Figure S4. PARP-1 subcellular distribution in TNBC and luminal BC patients' CTCs. (**A**) Percentage of early and metastatic TNBC and luminal patients with either nuclear or cytoplasmic expression of PARP-1 in their CTCs; (**B**) Percentage of CTCs with cytoplasmic or nuclear PARP-1 localization in early and metastatic TNBC and luminal patients.

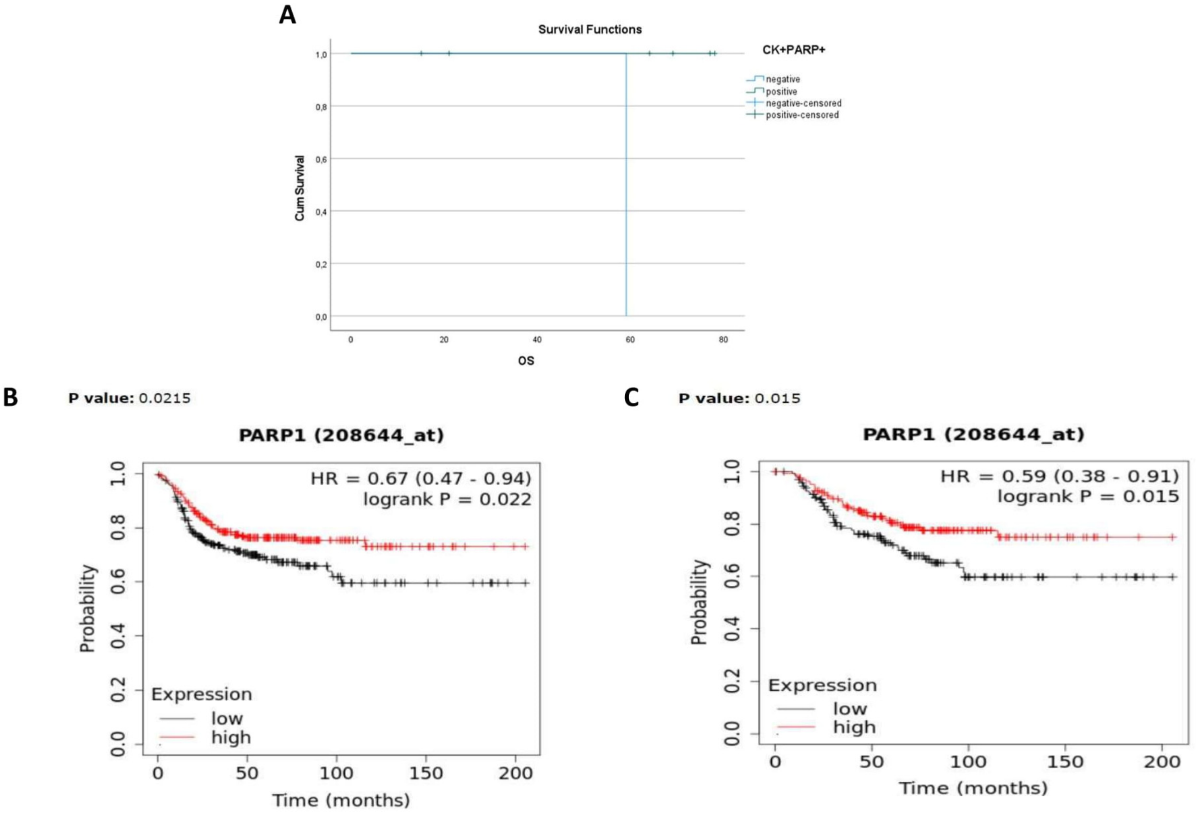

Figure S5. PARP-1 expression and clinical outcome. (**A**) Kaplan–Meier survival curve for CK-positive BC patients in respect to the CK^+PARP^+ phenotype (log-rank *p* = *0.046*). (**B**) Correlation of PARP-1 expression with Disease Free Interval (DFI) in patients with basal-like BC using KMplot (https://kmplot.com/analysis/). (**C**) Correlation of PARP-1 expression with OS in patients with basal-like BC using KMplot (https://kmplot.com/analysis/, accessed on 27 September 2021).

Table S1. Statistical analysis and *p* values for the different PARP-1 phenotypes and localizations identified in the corresponding graphs.

Figure	PARP expression	Test	*p* Values
1a	CK+PARP+	Chi-square	0.156
1a	CK+PARP-	Chi-square	0.834
1b	CK+PARP+	Chi-square	0.175
1b	CK+PARP-	Chi-square	0.491
1c	CK+PARP+	Mann-Whitney	0.701
1c	CK+PARP-	Mann-Whitney	0.701
1d	CK+PARP+	Mann-Whitney	0.860
1d	CK+PARP-	Mann-Whitney	0.860
S3a	CK+PARP+	Chi-square	0.232
S3a	CK+PARP-	Chi-square	0.755
S3b	CK+PARP+	Kruskal-Wallis	0.853
3a	Nuclear	Chi-square	0.951
3a	Cytoplasmic	Chi-square	**0.024**
3b	Nuclear	Mann-Whitney	0.230
3b	Cytoplasmic	Mann-Whitney	0.093
S4a	Nuclear	Chi-square	0.929
S4a	Cytoplasmic	Chi-square	**0.035**
S4b	Nuclear	Kruskal-Wallis	0.661
S4b	Cytoplasmic	Kruskal-Wallis	0.397

Table S2. Statistical analysis and *p* values for the identified mutations in the corresponding graphs.

Figure	Mutations	Test	*p* Values
4a	M1	Chi-square	0.321
4a	M2	Chi-square	0.363
4a	M3	Chi-square	0.054
4a	M4	Chi-square	0.857
4a	M5	Chi-square	0.507
4a	M6	Chi-square	0.908
4a	M7	Chi-square	**0.030**
4a	M8	Chi-square	0.507
4a	M9	Chi-square	0.777
4b	M1	Chi-square	0.363
4b	M2	Chi-square	0.725
4b	M3	Chi-square	0.209
4b	M4	Chi-square	0.945
4b	M5	Chi-square	0.159
4b	M6	Chi-square	0.521
4b	M7	Chi-square	**0.014**
4b	M8	Chi-square	**0.002**
4b	M9	Chi-square	0.483
